# Colonic expression of glutathione *S*-transferase alpha 4 and 4-hydroxynonenal adducts is correlated with the pathology of murine colitis-associated cancer

**DOI:** 10.1016/j.heliyon.2023.e19815

**Published:** 2023-09-04

**Authors:** Chunhua Ma, Zhanhu Zhang, Tianqi Li, Yumei Tao, Guoxiang Zhu, Lili Xu, Yuanyuan Ju, Xu Huang, Jinyun Zhai, Xingmin Wang

**Affiliations:** a Nantong Institute of Genetics and Reproductive Medicine, Affiliated Maternity and Child Healthcare Hospital of Nantong University, Nantong, China; b Department of Pathology, Affiliated Maternity and Child Healthcare Hospital of Nantong University, Nantong, China; c Department of Medical Experimental Technology, Nantong University Xinglin College, Nantong, China

**Keywords:** Colitis-associated cancer, 4-Hydroxynonenal, Glutathione *S*-Transferase alpha 4, AOM/DSS, Inflammatory cytokines

## Abstract

Chronic inflammation-induced oxidative stress is an important driving force for developing colitis-associated cancer (CAC). 4-hydroxynonenal (4-HNE) is a highly reactive aldehyde derived from lipid peroxidation of ω-6 polyunsaturated fatty acids that contributes to colorectal carcinogenesis. Glutathione *S*-transferase alpha 4 (Gsta4) specifically conjugates glutathione to 4-HNE and thereby detoxifies 4-HNE. The correlation of these oxidative biomarkers with the pathological changes in CAC is, however, unclear. In this study, we investigated the expression of Gsta4 and 4-HNE adducts in azoxymethane/dextran sulfate sodium (AOM/DSS)-induced murine CAC, and analyzed the correlations of 4-HNE and Gsta4 with inflammatory cytokines and the pathological scores in the colon biopsies. Real-time quantitative PCR showed that expression of IL6, TNFα, and Gsta4 sequentially increased in colon tissues for mice treated with DSS for 1, 2, and 3 cycles, respectively. Moreover, immunohistochemical staining showed remarkably increased expression of 4-HNE adducts, Gsta4, TNFα, and IL6 in the colon biopsies after 3 cycles of DSS treatment. Correlation analysis demonstrated that 4-HNE adducts in the colon biopsies were positively correlated with Gsta4 expression. Additionally, the expression of Gsta4 and 4-HNE adducts were strongly correlated with the pathological changes of colon, as well as the expression of TNFα and IL6 in colon tissues. These results provide evidence for the association of oxidative biomarkers Gsta4 and 4-HNE with the pathological changes of CAC and may help developing novel histopathological biomarkers and prevention targets for CAC.

## 1 Introduction

Colorectal cancer (CRC) is the third most common cancer type and the second leading cause of cancer-related death worldwide, which causes colossal medical and economic burden [[1], [2], [3]]. For the past decades, the incidence of CRC has declined over 30% for people aged ≥50 in developed countries like the United States. This is largely attributed to the CRC screening program started in 1990s [4]. In contrast, the overall CRC incidence is predicted to increase sharply for the next decade in many other countries including China, Croatia, UK, and Japan [5]. Notably, the incidence of early-onset CRC (*i.e.*, CRC diagnosed in patients younger than 50 years of age) and the related death are significantly increasing [6]. As such, the American Cancer Society has lowered the screening age from 50 to 45 [7].

The risk factors of CRC include age, genetic background, obesity, lifestyles, gut microbiota as well as chronic inflammation [[8], [9], [10]]. Of these, chronic inflammation is an important risk factor for developing CRC. In comparison with 3–5% life-time risk of developing sporadic colorectal cancer in general population, patients with inflammatory bowel diseases (*e.g.*, ulcerative colitis and Crohn's disease) are associated with more than 3-fold increased risk of CRC, known as colitis-associated cancer (CAC) [11]. CAC shares many molecular features with sporadic CRC, however, unlike sporadic CRC, the development of CAC does not follow the adenoma-carcinoma sequence —a common feature of sporadic CRC [10,12]. Chronic inflammation can generate various inflammatory cytokines that activate carcinogenic signaling pathways and induce epigenetic modification leading to CAC [13]. Of these, tumor necrosis factor alpha (TNFα) and interleukin 6 (IL6) have long been linked to the development of CAC [[13], [14], [15], [16]]. TNFα promotes CAC via the TNF receptor superfamily 1b (*Tnfrsf1b*)-dependent NF-κB activation in different murine CAC models [14,17]. Similarly, blocking *Tnfrsf1a*, another key member of the TNF receptor superfamily, also reduces mucosal damage, inflammation, and tumor formation in an azoxymethane/dextran sulfate sodium (AOM/DSS)-induced CAC model [18]. IL6, on the other hand, is a NF-κB-regulated cytokine that contributes to the development of CAC via the IL6-Stat3 cascade [15,19].

In addition to inflammatory cytokines, chronic inflammation can generate mutagenic reactive oxygen/nitrogen species (ROS/RNS) that cause DNA damage and mutations driving neoplastic transformation [13,[20], [21], [22]]. 4-hydroxynonenal (4-HNE) is a lipid peroxidation byproduct of ω-6 polyunsaturated fatty acids (PUFAs) that is an important carcinogenic signaling inducer [23]. 4-HNE acts as a mutagen leading to double-stranded DNA breaks, mutations, aneuploidy, and neoplastic transformation via the bystander effect [24,25]. 4-HNE can readily form adducts with DNA, RNA, and proteins. These adducts were enriched in human colorectal cancer and murine CAC [24,26,27]. However, whether the accumulation of 4-HNE adducts is associated with the pathological changes as well as the expression of inflammatory cytokines in CAC is still unclear.

Glutathione *S*-transferase alpha 4 (GSTA4) is a phase II detoxifying enzyme that specifically metabolizes 4-HNE through catalyzing the conjugation of 4-HNE to glutathione. GSTA4 is associated with certain types of human cancers, for example, *GSTA4* mutation significantly increases susceptibility to human skin cancer and chemical-induced murine skin cancer, suggesting a protective role of GSTA4 against skin cancer [28]. GSTA4 is overexpressed in several types of human cancer including breast, kidney, liver, and colorectal cancer [29]. Expression of GSTA4 in human CRC cells is regulated by oncogenic transcription factor AP-1 [26]. Most recently, we demonstrated that overexpression of GSTA4 promoted proliferation and chemoresistance in human CRC cells [30].

AOM/DSS-induced murine CAC model is a well-accepted animal model for CRC research because it shares many histopathological and molecular features with human CRC. For example, tumors in AOM/DSS-induced adenocarcinomas often located in the distal colon of mice, which is similar to human CRC that frequently occurred in the left colon and rectum. In addition, AOM/DSS-induced CAC shows activation of many oncogenic pathways including Wnt/β-catenin, notch, hedgehog, and TGFβ that are often activated in human CRC [31]. Most importantly, combination of AOM and DSS can induce CAC in a short time period, which provides a time-saving and relatively inexpensive model for mimicking human CAC. AOM is unable to directly damage DNA, however, AOM can be metabolized by cytochrome P450 in the liver to form methylazoxymethanol that is further broken down to methyldiazonium, a highly reactive chemical, leading to DNA alkylation and mutations [32]. DSS is a negatively charged polysaccharide that can be metabolized by intestinal bacteria. DSS damages intestinal barrier, which allows luminal bacteria invade the mucosa leading to inflammation [33].

In the present study we investigated the expression of Gsta4, 4-HNE adducts, and inflammatory cytokines TNFα and IL6 in the AOM/DSS-induced CAC model. Furthermore, we analyzed the correlation of oxidative stress markers Gsta4 and 4-HNE adducts with the expression of inflammatory cytokines TNFα and IL6, as well as pathological changes. We found that Gsta4, 4-HNE adducts, TNFα, and IL6 were strongly expressed in the colorectal biopsies from AOM/DSS-treated mice and correlated with the pathological changes. Notably, the expression of Gsta4 and 4-HNE adducts is strongly correlated with the expression of TNFα and IL6. These findings indicate that Gsta4 and 4-HNE adducts, the oxidative biomarkers, are correlated with the inflammatory biomarkers and may have diagnostic value for CAC.

## 2 Results

### 2.1 AOM/DSS-induced colitis-associated cancer

**Fig. 1:**
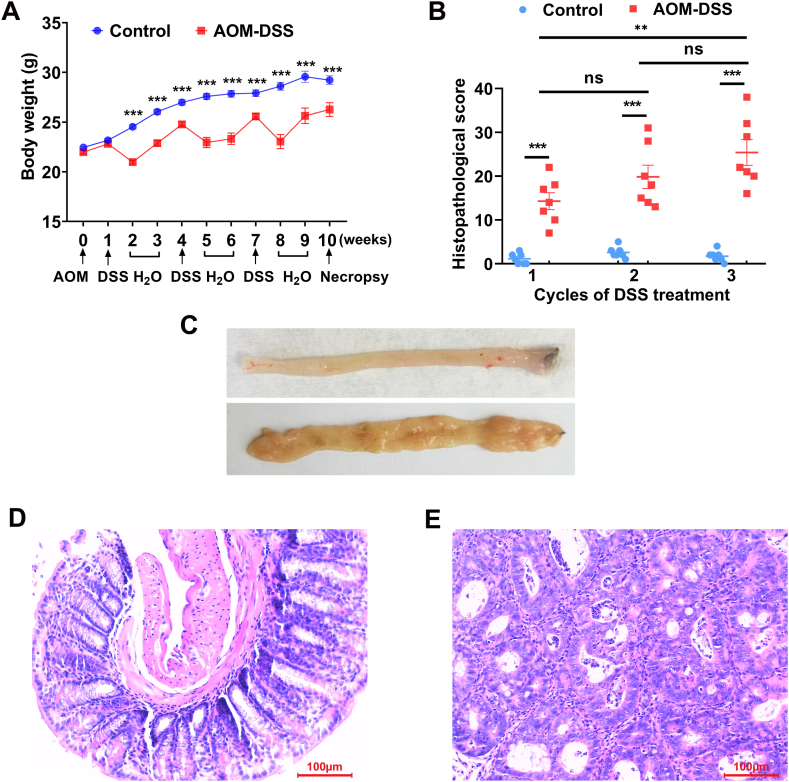
**AOM/DSS-induced murine CAC model.** (A) Body weight loss is seen following DSS treatment. (B) Histopathological scores of large intestines. ns, not significant; **P < 0.01, and ***P < 0.001 vs control group, n = 7 per time point per group. (C) Representative large intestines from untreated mouse (*Upper*) and AOM/DSS-treated mouse (*Lower*). (D) H & E staining for colon biopsy from representative untreated mouse. (E) H & E staining for colon biopsy from representative AOM/DSS-treated mouse showing carcinoma in situ. All photomicrographs are ×20.

**Supplementary Figure S1:**
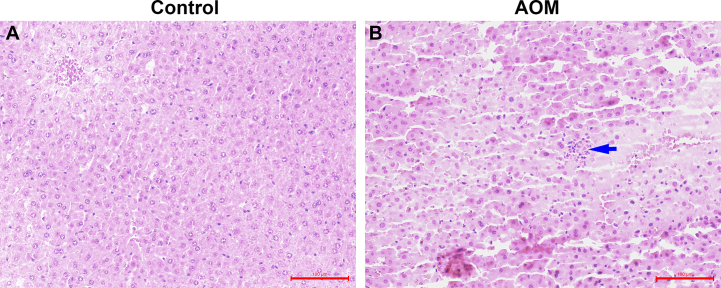
**AOM rarely induces lesions in liver**. (A) H & E staining for liver biopsy from untreated mouse. (B) H & E staining shows rare hepatocyte necrosis in liver biopsy from AOM-treated mouse 1 week after AOM injection (Blue arrow). Scale bar: 100µm.

**Supplementary Figure S2:**
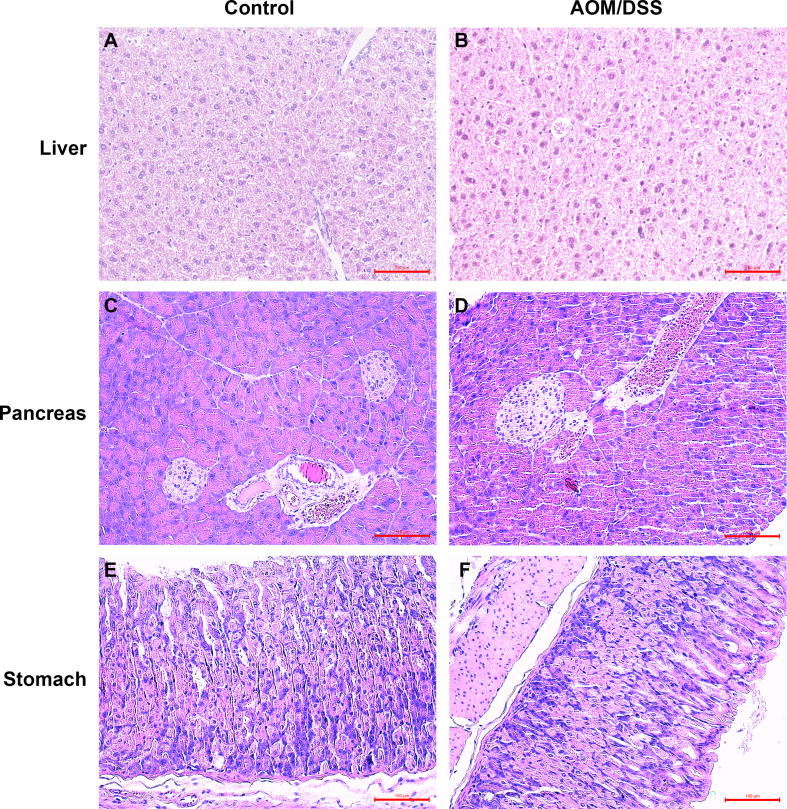
**AOM/DSS treatment does not cause injury to organs outside of intestines.** (A and B) Representative H & E staining for liver biopsies from mice treated with sham or AOM and 3 cycles of DSS. (C and D) Representative H & E staining for pancreatic biopsies from mouse treated with sham or AOM and 3 cycles of DSS. (E and F) Representative H & E staining for stomach biopsies from mouse treated with sham or AOM and 3 cycles of DSS. Scale bar: 100µm.

To assess the correlation of oxidative biomarkers 4-HNE and Gsta4 with the pathological changes in CAC, we established an AOM/DSS-induced murine CAC model. No body weight change was noted one week after AOM injection compared to untreated control group. However, body weight loss was observed for mice treated with DSS (Fig. 1A). In addition, diarrhea and fecal blood presented in AOM/DSS-treated mice. Histopathological evaluation for colon biopsies showed no inflammation for control mice that were given saline throughout the experiment (Fig. 1B, C, and D), however, pathological scores significantly increased for mice treated with AOM and 3 cycles of DSS compared to mice treated with 1 cycle of DSS (Fig. 1B, C, and E, *P* < 0.01). Following 3 cycles of DSS treatment, severe inflammation and carcinomas presented in all mice treated with AOM/DSS (Fig. 1C and E). Finally, pathological evaluation showed minor hepatocyte necrosis in the liver biopsies from 2 mice (2 out of 28 mice) 1 week after AOM injection (Supplementary Fig. S1), otherwise, no inflammation or injury was noted in other alimentary organs including liver, pancreas, and stomach after each cycle of DSS treatment (Supplementary Fig. S2).

### 2.2 Serum Gsta4 and 4-HNE adducts are less associated with the pathology of CAC

**Supplementary Figure S3:**
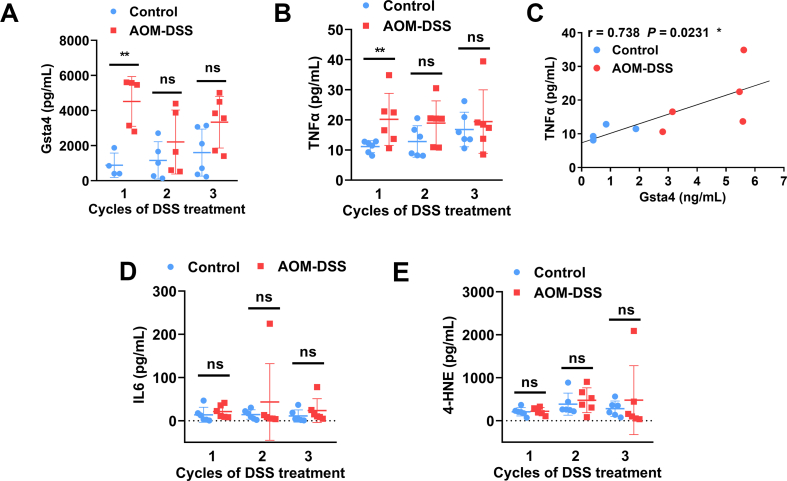
**Serum Gsta4 and 4-HNE are less associated with the pathology of AOM/DSS-induced CAC.** (A and B) ELISA shows transiently increased serum Gsta4 (A) and TNFα (B) after one cycle treatment with DSS. No increased serum Gsta4 and TNFα are noted after the 2^nd^ and 3^rd^ cycles of DSS treatment. (C) Serum Gsta4 is correlated with TNFα following one cycle treatment with DSS. (D and E) No remarkable change is noted for serum IL6 and 4-HNE throughout the experiment. At least 4 mice per group were used for ELISA experiments based on the availability of serum. **P < 0.01 compared to control; ns, not significant.

Because serum Gsta4 transiently increased at the early stage in a commensal bacteria-induced CRC model [26], we initially analyzed serum Gsta4 and 4-HNE adducts and compared them with serum levels of TNFα and IL6, two major inflammatory cytokines, in AOM/DSS-induced CAC mice. We found that serum Gsta4 and TNFα transiently increased in mice after one cycle of DSS treatment compared to untreated mice (Supplementary Figs. S3A and B). In addition, serum Gsta4 was correlated with TNFα level following one cycle of DSS treatment (Supplementary Fig. S3C, *r* = 0.738, *P* < 0.05). No remarkably increased serum Gsta4 and TNFα were seen after the 2nd and 3rd cycles of DSS treatment compared to control mice (Supplementary Figs. S3A and B). In contrast, no significantly increased serum IL6 and 4-HNE were noted for mice treated with AOM/DSS compared to untreated mice throughout the experiment (Supplementary Figs. S3D and E). These results imply that serum Gsta4 and 4-HNE adducts may not be associated with the pathological changes in AOM/DSS-induced CAC.

### 2.3 Colorectal expression of Gsta4 is correlated with the expression of inflammatory cytokines

**Fig. 2:**
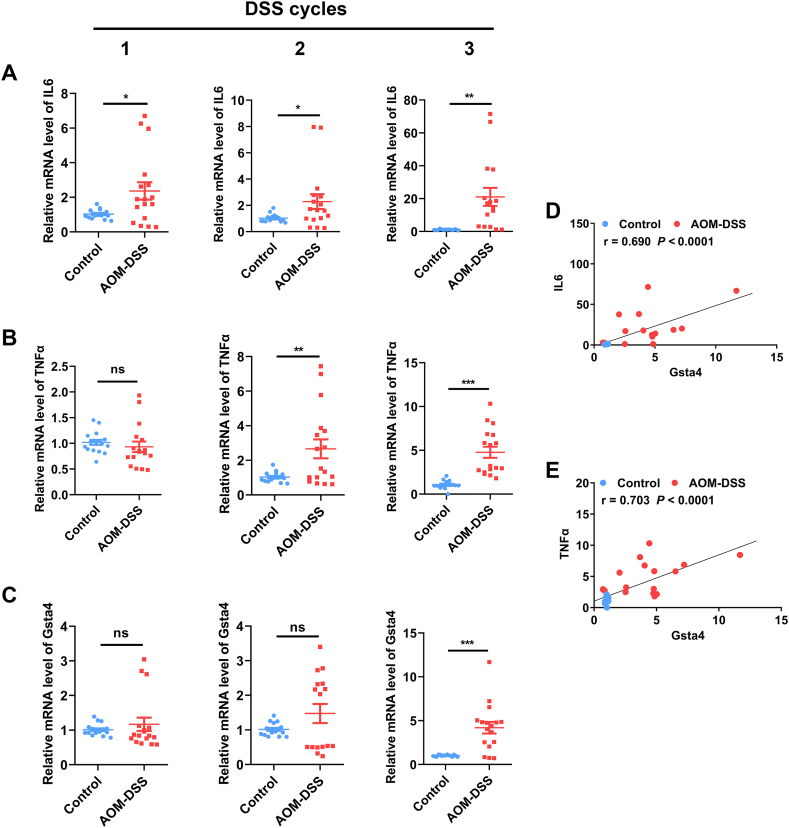
**Gene expression of Gsta4, IL6, and TNFα in colorectal biopsies.** (A–C) Quantitative RT-PCR analyses show sequentially increased gene expression of IL6 (A), TNFα (B), and Gsta4 (C) in the colorectal biopsies from mice treated with DSS (*Red*) compared to untreated mice (*Blue*). Data are derived from 7 mice for each group with at least 2 repeats of qRT-PCR. ***P < 0.001, **P < 0.01, and *P < 0.05 compared to control. (D and E) The expression of Gsta4 is strongly correlated with the expression of IL6 (D) and TNFα (E) in colorectal biopsies.

Because serum Gsta4 and 4-HNE adducts were inadequately associated with the pathological changes and serum inflammatory cytokines, we determined gene expression of Gsta4 and inflammatory cytokines in colon tissues. Quantitative RT-PCR showed sequentially increased expression of IL6 and TNFα after 1 and 2 cycles of treatment with DSS, respectively, compare to control mice (Fig. 2A and B). In contrast, significantly increased colorectal expression of Gsta4 was only seen after 3 cycles of DSS treatment (Fig. 2C, *P* < 0.01). Similarly, gene expression of IL6 and TNFα also significantly increased in colon biopsies from mice treated with 3 cycles of DSS compared to untreated mice (Fig. 2A and B). We next analyzed the correlations of Gsta4 expression with the expression of IL6 and TNFα for mice treated with 3 cycles of DSS. As shown in Fig. 2D and E, the colorectal expression of Gsta4 was strongly correlated with the expression of IL6 (*r* = 0.690, *P* < 0.001) and TNFα (*r* = 0.703, *P* < 0.001), respectively, in colon biopsies.

### 2.4 Colorectal expression of Gsta4 is correlated with the pathological scores in CAC

**Fig. 3:**
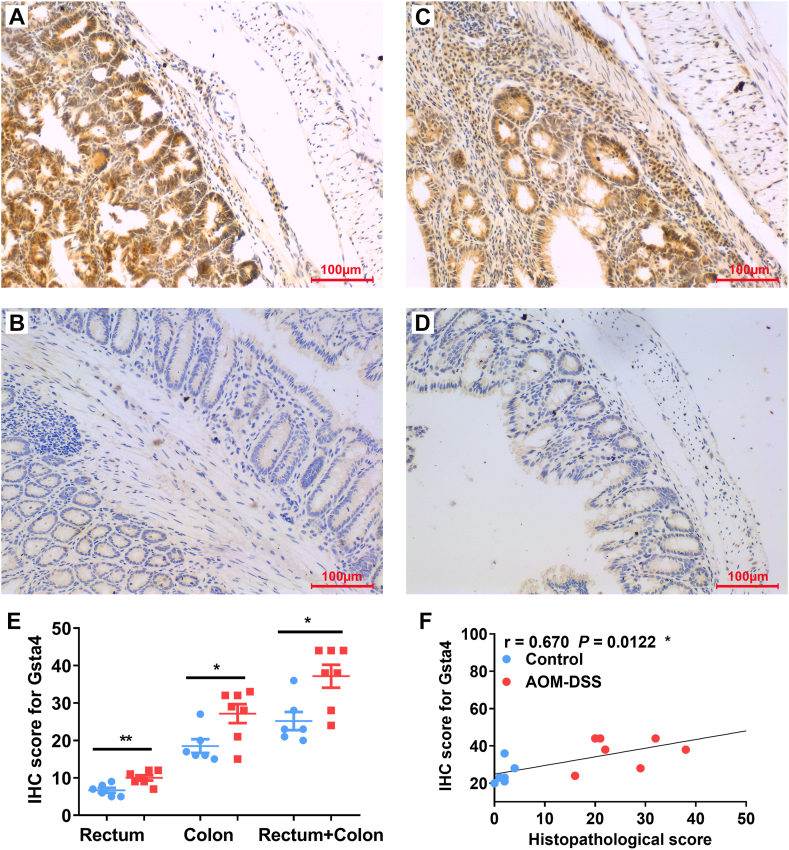
**Gsta4 expression in colorectal biopsies is correlated with the pathological changes.** (A and B) Representative IHC staining for Gsta4 (*brown*) in the rectum from mouse treated with 3 cycles of DSS (A) compared to untreated mouse (B). (C and D) Representative IHC staining for Gsta4 in the colon biopsies from mouse treated with 3 cycles of DSS (C) compared to untreated mouse (D). (E) IHC scores for Gsta4 staining in the biopsies from rectum, colon, and large intestine (Rectum + Colon). **P < 0.01 and *P < 0.05 for AOM/DSS (*Red*) vs control (*Blue*), n = 7 for each group. (F) Gsta4 scores are correlated with the histopathological scores in colorectal biopsies. All photomicrographs are ×20.

To confirm the results from qRT-PCR, biopsies from large intestine including proximal, middle, and distal colons and rectum were immunohistochemically stained for Gsta4. Gsta4 was strongly stained in rectal biopsies from mice treated with 3 cycles of DSS compared to minor staining in rectal biopsies from untreated mice (Fig. 3A, B, and E). Similarly, colon biopsies were also positively stained for Gsta4 in mice treated with 3 cycles of DSS compared to negative staining in control mice (Fig. 3C, D, and E). IHC staining scores for large intestine (Total scores of proximal, middle, and distal colons and rectum) were significantly correlated with the histopathological scores (Fig. 3F, *r* = 0.670, *P* < 0.05), suggesting that Gsta4 expression in the colorectal biopsies may be a potential biomarker for CAC.

### 2.5 AOM/DSS-treatment generates 4-HNE adducts in colorectal tissues

**Fig. 4:**
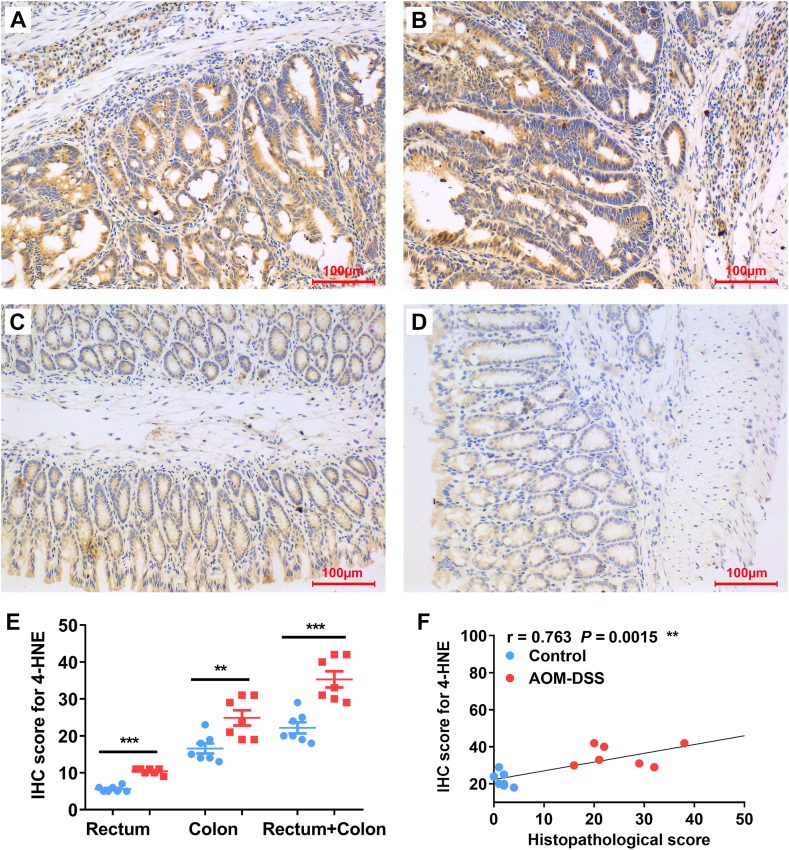
**4-HNE adducts in colorectal biopsies are correlated with the pathological changes.** (A and B) Representative IHC staining for 4-HNE adducts (*brown*) in the rectum (A) and colon (B) from mouse treated with 3 cycles of DSS. (C and D) Representative IHC staining for 4-HNE adducts in the biopsies of rectum (C) and colon (D) from untreated mouse. (E) IHC scores for 4-HNE staining in the biopsies from rectum, colon, and large intestine (Rectum + Colon). ***P < 0.001 and **P < 0.01 for AOM/DSS (*Red*) *vs* control (*Blue*), n = 7 for each group. (F) IHC scores for 4-HNE adducts are strongly correlated with the histopathological scores in colorectal biopsies. All photomicrographs are ×20.

Previous studies have shown that 4-HNE adducts were enriched in colorectal cancer [24,26]. To determine whether AOM/DSS treatment induces 4-HNE production, we stained 4-HNE adducts for colorectal biopsies. IHC staining showed that 4-HNE adducts remarkably increased in the rectal (Fig. 4A) and colonic (Fig. 4B) biopsies from mice treated with 3 cycles of DSS compared to minor staining in biopsies from untreated mice (Fig. 4C and D for rectal and colonic biopsies, respectively). IHC staining scores of colorectal biopsies significantly increased for mice treated with 3 cycles of DSS compared to untreated mice (Fig. 4E). Moreover, the total scores of IHC for large intestine (Sum of colon and rectum) were significantly correlated with the pathological scores (Fig. 4F, *r* = 0.763, *P* < 0.01).

### 2.6 TNFα and IL6 expression in the colorectal biopsies of CAC

**Fig. 5:**
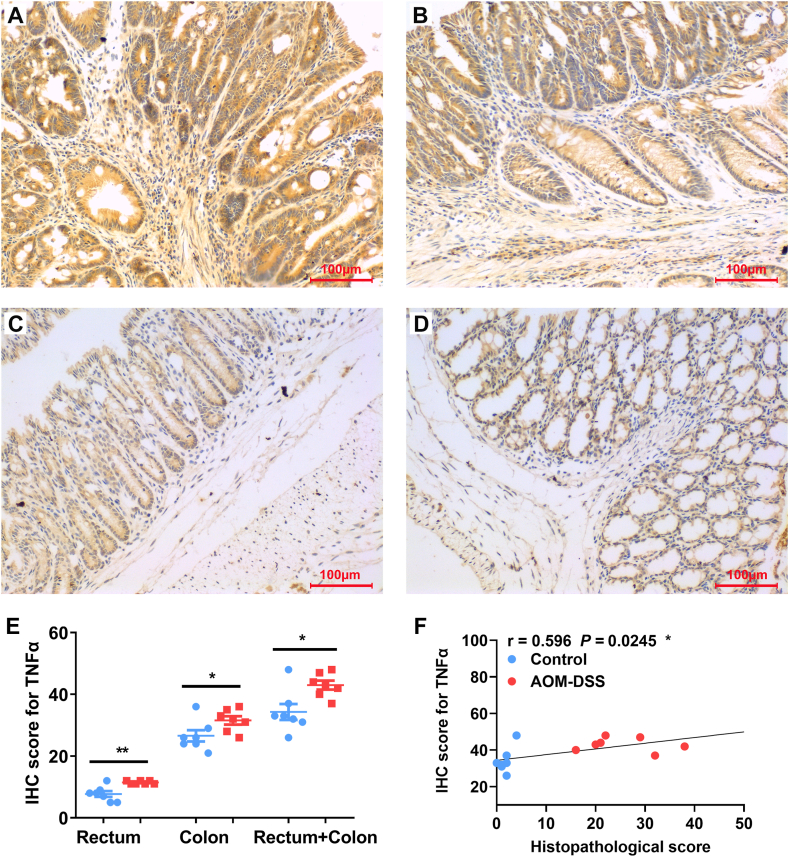
**TNFα expression in colorectal biopsies.** (A and B) Representative IHC staining for TNFα (*brown*) in the biopsies of rectum (A) and colon (B) from mouse treated with 3 cycles of DSS. (C and D) Representative IHC staining for TNFα in the biopsies of rectum (C) and colon (D) from untreated mouse. (E) IHC scores for TNFα staining in the biopsies from rectum, colon, and large intestine (Rectum + Colon). **P < 0.01 and *P < 0.05 for AOM/DSS (*Red*) *vs* control (*Blue*), n = 7 for each group. (F) The expression of TNFα in colorectal biopsies are correlated with the histopathological scores. All photomicrographs are ×20.

**Fig. 6:**
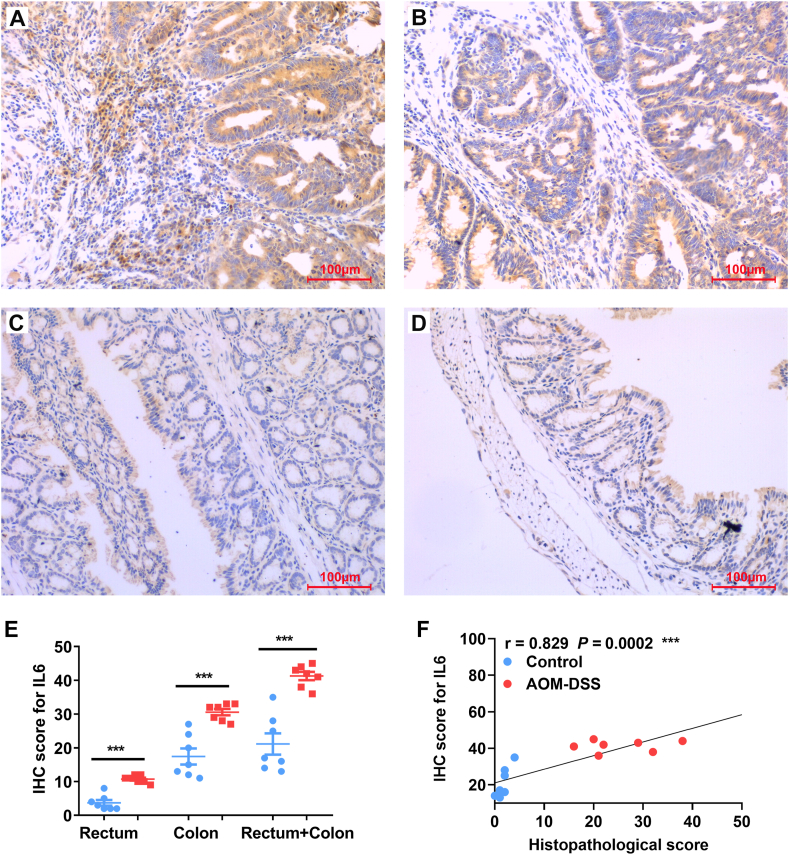
**IL6 expression in colorectal biopsies.** (A and B) Representative IHC staining for IL6 (*brown*) in the biopsies of rectum (A) and colon (B) from mouse treated with 3 cycles of DSS. (C and D) Representative IHC staining for IL6 in the biopsies of rectum (C) and colon (D) from untreated mouse. (E) IHC scores for IL6 staining in the biopsies from rectum, colon, and large intestine (Rectum + Colon). ***P < 0.001 for AOM/DSS (*Red*) *vs* control (*Blue*), n = 7 for each group. (F) The expression of IL6 in colorectal biopsies are strongly correlated with the histopathological scores. All photomicrographs are ×20.

Because we did not observe increased serum TNFα and IL6 in mice after the 2nd and 3rd cycles of treatment with DSS compared to untreated mice, we next determined the expression of TNFα and IL6 in colorectal biopsies using IHC staining. As expected, TNFα production remarkably increased in rectal (Fig. 5A and E) and colonic biopsies (Fig. 5B and E) from mice treated with 3 cycles of DSS compared to untreated mice (Fig. 5C, D, and E). The overall IHC staining scores for TNFα production in whole large intestine were correlated with the pathological scores (Fig. 5F, *r* = 0.596, *P* < 0.05). Similarly, IL6 production also remarkably increased in the biopsies of rectum (Fig. 6A and E) and colons (Fig. 6B and E) from AOM/DSS-treated mice compared to untreated mice (Fig. 6C, D, and E). The IHC staining scores for IL6 production in the large intestinal biopsies were significantly correlated with the pathological scores (Fig. 6F, *r* = 0.829; *P* < 0.001).

### 2.7 Rectal Gsta4 and 4-HNE adducts are correlated with inflammatory cytokines

**Fig. 7:**
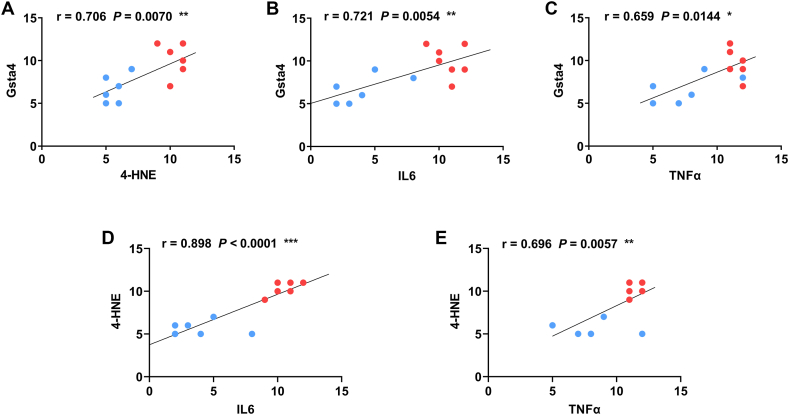
**Correlation analyses for 4-HNE adducts, Gsta4, TNFα, and IL6 in the rectal biopsies.** Significant correlations are observed for Gsta4 *vs* 4-HNE adducts (A), Gsta4 *vs* IL6 (B), Gsta4 *vs* TNFα (C), 4-HNE adducts *vs* IL6 (D), and 4-HNE adducts *vs* TNFα expression (E) in the rectal biopsies.

**Supplementary Figure S4:**
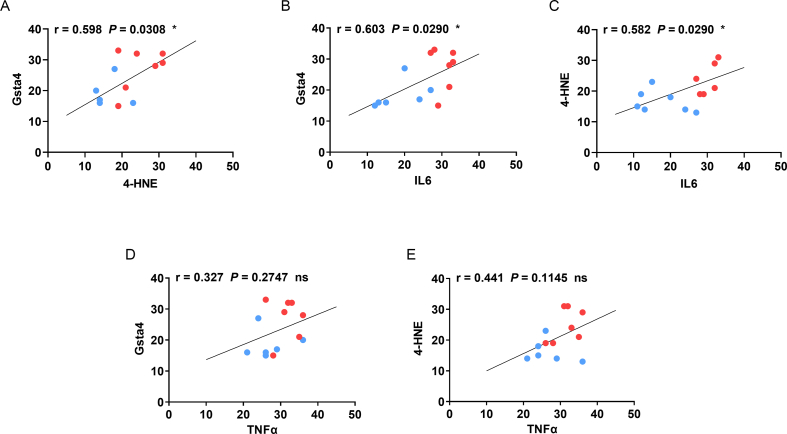
**Correlation analyses for 4-HNE adducts, Gsta4, TNFα, and IL6 in the colon biopsies.** Significant correlations exist between the IHC scores of Gsta4 *vs* 4-HNE adducts (A), Gsta4 *vs* IL6 (B), and 4-HNE adducts *vs* IL6 (C) in the colon biopsies. In contrast, no significant correlations are seen for IHC scores of TNFα *vs* Gsta4 (D) and TNFα *vs* 4-HNE adducts (E) in the colon biopsies. *Red dots*, AOM/DSS; *Blue dots*, control.

Finally, we analyzed the correlations of IHC scores for Gsta4, 4-HNE adducts, and inflammatory cytokines. Gsta4 staining scores for colon biopsies (Sum of proximal, middle, and distal colons) were correlated with the scores of 4-HNE adducts (Supplementary Fig. S4A; *r* = 0.598, *P* < 0.05). In addition, both Gsta4 and 4-HNE adduct scores were correlated with IL6 scores in the colon biopsies (Supplementary Figs. S4B and C; *r* = 0.603, *P* < 0.05 for Gsta4 *vs* IL6 and *r* = 0.582, *P* < 0.05 for 4-HNE adducts *vs* IL6, respectively). In contrast, neither Gsta4 nor 4-HNE adduct scores were significantly correlated with TNFα scores in the colon biopsies (Supplementary Figs. S4D and E; *r* = 0.327, *P* = 0.27 for Gsta4 vs TNFα and *r* = 0.441, *P* = 0.11 for 4-HNE adducts vs TNFα, respectively). In the rectal biopsies, the IHC staining scores of Gsta4 were significantly correlated with the scores of 4-HNE adducts (Fig. 7A, *r* = 0.706, *P* < 0.01). Notably, Gsta4 staining scores were significantly correlated with scores of IL6 (Fig. 7B, *r* = 0.721, *P* < 0.01) and TNFα (Fig. 7C, *r* = 0.659, *P* < 0.05) in the rectal biopsies. Similarly, IHC staining scores for 4-HNE adducts were also significantly correlated with scores of IL6 (Fig. 7D, *r* = 0.898, *P* < 0.001) and TNFα (Fig. 7E, *r* = 0.696, *P* < 0.01) in the rectal biopsies. These results suggest that Gsta4 and 4-HNE adducts are strongly correlated with inflammatory cytokines in the rectal biopsies and thus may have potential to serve as biomarkers for CAC.

## 3 Discussion

Inflammation-induced oxidative damage to the genome is linked to the development of colitis-associated cancer (CAC). 4-HNE is a byproduct of ω-6 polyunsaturated fatty acid peroxidation that causes DNA damage, G2/M arrest, chromosomal instability, and neoplastic transformation [24,25,34]. Glutathione *S*-transferase alpha 4 (GSTA4) is a phase II detoxifying enzyme that specifically metabolizes 4-HNE. Previous studies have shown that GSTA4 and 4-HNE adducts are highly expressed in human colorectal adenomas and carcinomas as well as in murine CRC models [26,27]. Whether or not the overexpression of oxidative biomarkers such as Gsta4 and 4-HNE adducts are associated with the pathological changes of CRC is, however, unclear. In this study we utilized an AOM/DSS-induced CAC murine model to investigate the correlations of Gsta4 and 4-HNE adducts with the expression of inflammatory cytokines as well as pathological changes during colitis-associated cancer development. We found that serum Gsta4 was correlated with TNFα at early stage of colitis whereas no correlation was found at later stage of carcinogenesis. In addition, the expression of Gsta4 and 4-HNE adducts in colorectal biopsies was correlated with the degree of pathological changes and the expression of TNFα and IL6. These results suggest that oxidative biomarkers 4-HNE adducts and Gsta4 are strongly correlated with the inflammatory biomarkers in CAC. As 4-HNE initiates CRC via mediating microbiota-induced bystander effect and Gsta4 promotes CRC cell proliferation [24,25,30], the results from the current study further support the tumor-promoting roles of 4-HNE and Gsta4.

GSTA4, as an antioxidative biomarker, is strongly expressed in the colon biopsies from ulcerative colitis patients compared to healthy controls [35]. Because Gsta4 specifically conjugates glutathione to 4-HNE and thus detoxifies 4-HNE, one might expect decreased 4-HNE adducts when Gsta4 is overexpressed, in other words, 4-HNE adducts might be negatively correlated with Gsta4. In contrast, we observed that both Gsta4 and 4-HNE adducts remarkably increased in the colon biopsies from AOM/DSS-induced CAC in this study. This may be due to that 4-HNE can induce Gsta4 expression in mouse colonic epithelial cells [26]. Notably, in a Gsta4-deficient mouse model, 4-HNE levels did dramatically increase in liver, whereas 4-HNE levels remain low in the heart tissues compared to wild-type mice, suggesting Gsta4-independent 4-HNE accumulation [36]. Indeed, inactivation of GSTA4 in colon cancer cells had very limited effect on the 4-HNE detoxification [30]. Interestingly, unlike inflammatory cytokines that appeared in colon tissues as early as after 1 cycle treatment with DSS, Gsta4 expression only increased in mice treated with 3 cycles of DSS (Fig. 2). This is consistent with previous results showing that GSTA4 specifically expressed in adenomas and carcinomas, not in normal and hyperplastic colons [26]. These findings support that oxidative tumor microenvironment substantially generates 4-HNE that, in turn, induces Gsta4 expression—a consequence of 4-HNE overloading in the oxidative microenvironment of tumor.

4-HNE can be generated by different types of cells depending on the stage of CAC development. These cells may be immune cells in the early inflammation stage and tumor microenvironment as well as cancer cells in the established tumors. We previously reported that abnormally activated macrophages can produce 4-HNE through the cyclooxygenase-2 (COX-2)-dependent manner, which contributed to the initiation of commensal bacteria-induced CAC [24,37]. COX-2 catalyzes enzymatic lipid peroxidation and links inflammation to CRC. In addition, chronic inflammation generates substantial ROS that trigger non-enzymatic lipid peroxidation leading to 4-HNE production [38]. Unlike normal cells, rapidly proliferating cancer cells usually have altered fatty acid uptake and metabolism [39,40], which may cause abnormal accumulation of 4-HNE in cancer. Recent studies found that, under acidic tumor environment, excessive accumulation of both ω-3 and ω-6 PUFAs enhances lipid peroxidation in cancer cells [41]. These studies support that accumulation of 4-HNE in the colorectal cancer may be derived from excessive lipid metabolism.

4-HNE is one of clastogenic factors that induces gene mutations and chromosomal instability [24,25,34]. 4-HNE, on the other hand, is a signaling inducer in carcinogenesis [23]. 4-HNE is capable of blocking NF-kB activation and thus reduces production of TNFα and IL-6 in monocytes and mast cells [42,43]. We found increased serum TNFα only at the early stage of colitis. This might be due to NF-kB inhibition by gradually increased 4-HNE that, consequently, blocks production of TNFα and IL6. Investigation of plasma 4-HNE in cancer patients is still lacking. In the present study we measured serum 4-HNE concentration using an ELISA kit that detects 4-HNE adducts, but not free 4-HNE, and we found no difference between the AOM/DSS and control groups (Supplementary Fig. S3E). Several studies have developed techniques for detecting 4-HNE or its metabolites in human plasma and urinary samples [44,45]. Future studies using these techniques to measure free 4-HNE in the plasma of cancer patients is strongly recommended to determine the association of 4-HNE with cancer.

Numerous studies have shown the diagnostic potential of glutathione *S*-transferases, particularly glutathione *S*-transferase pi, for colorectal cancer patients [46,47]. Alpha class glutathione *S*-transferases rapidly responds to liver injury and positively correlated with other diagnostic markers such as alanine aminotransferase (ALT) and aspartate aminotransferase (AST) [48,49]. However, few studies have assessed the value of GSTA4 as a biomarker for colorectal cancer so far. We observed increased plasma Gsta4 only after the first cycle of DSS treatment while no remarkably increased plasma Gsta4 after the 2nd and 3rd cycles of DSS treatment. This is similar to the previous findings in commensal bacteria-induced CAC using the *Il10*^−/−^ mouse model [26], and it might be due to the shorter half-life of alpha class GST as shown in patients with liver injury [49]. Coincidentally, Maffei and colleagues found that plasma glutathione *S*-transferase activity in CRC patients was comparable with the healthy controls and patients with adenomas, however, plasma catalase, glutathione reductase, superoxide dismutase, and clastogenic factors were significantly changed, either decreased or increased, in patients with CRC [50].

Although we showed that serum Gsta4 and 4-HNE adducts were less associated with the pathology of CAC, these results need further validation because of the small sample size and large variations in the ELISA results. As a major limitation of this study, we were unable to collect blood samples from all mice that were enrolled in the experiment due to technical difficulty leading to small sample size for serum study. Nonetheless, because our primary aim was to investigate the correlations of colonic expression of Gsta4 and 4-HNE adducts with the pathology of CAC, these negative results from serum studies have little impact on our conclusion. Large-scale studies focusing on blood samples from CAC patients are needed in the future.

Previous studies have shown that TNFα and IL6 increase in patients with ulcerative colitis [51], and deletion of TNFα or IL16 reduces CAC in murine models [15,18]. Although the average serum TNFα and IL6 levels in AOM/DSS-treated mice in the current study are close to the levels as previously reported [52], we observed only increased serum TNFα after the first treatment with DSS while no increased serum IL6 was seen for DSS-treated mice throughout the experiment. This is inconsistent with the findings reported by others in which they found liver injury along with increased serum IL6, IL1β, and TNFα after 1 cycle treatment with 3% DSS [53]. As shown in our results, we did not see DSS-induced organ injury or inflammation outside of intestines, meaning no systemic response that might affect serum inflammatory cytokines. Therefore, we speculate that lacking difference between AOM/DSS and control groups for serum IL6 might be due to the large inter-individual variability and/or absence of AOM/DSS-induced systemic responses in this study.

**Supplementary Figure S5:**
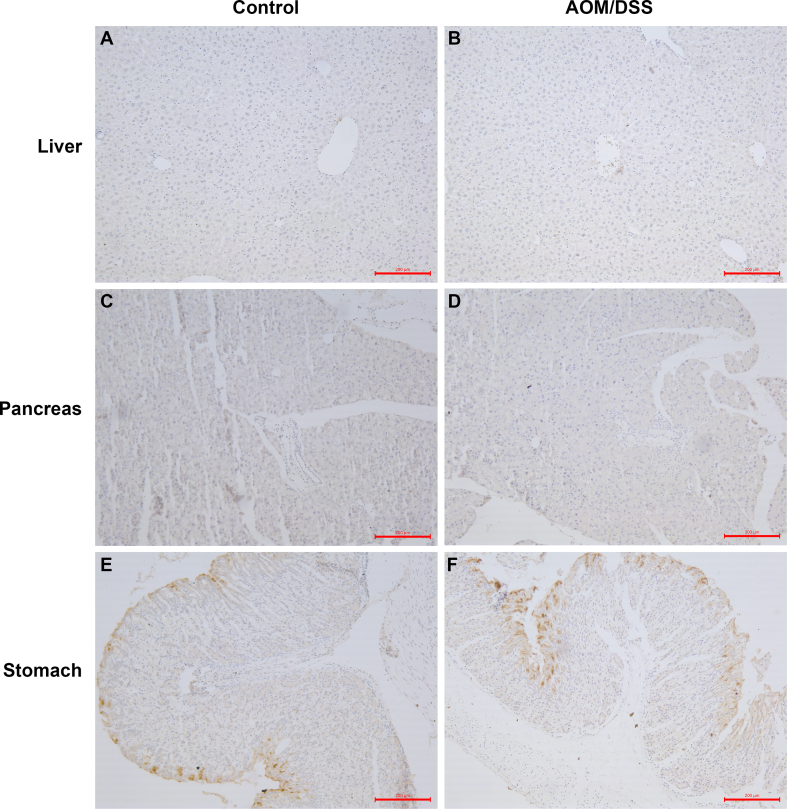
**AOM/DSS treatment does not generate 4-HNE adducts in liver, pancreas, and stomach.** (A and B) IHC staining for 4-HNE adducts in liver biopsies from mouse treated with sham or AOM and 3 cycles of DSS. (C and D) IHC staining for 4-HNE adducts in pancreatic biopsies from mouse treated with sham or AOM and 3 cycles of DSS. (E and F) IHC shows partially positive staining for 4-HNE adducts in the gastric foveolae for both control and AOM/DSS-treated mice. Scale bar: 200µm.

AOM/DSS were reported to specifically induce colitis and CRC, however, recent studies also showed inflammation outside of colon such as liver and kidney [[53], [54], [55]]. Duan and colleagues found that short-term treatment of BALB/C mice with 4% DSS in drinking water induced liver inflammation and hepatocyte necrosis along with increased inflammatory cytokines in liver tissues [54]. Other studies, however, showed no liver injury or inflammation for BALB/cKor1 mice treated with DSS compared to control [56]. Likewise, we did not find inflammation or injury in liver, stomach, and pancreas for mice treated with DSS in this study. These controversial findings may reflect mouse strain- and dose-dependency of AOM/DSS-induced CAC [57]. In addition, no detectable 4-HNE adducts was seen in liver and pancreas for mice treated with or without AOM/DSS, whereas gastric foveolae were partially stained for 4-HNE adducts for both control and AOM/DSS-treated mice (Supplementary Fig. S5). This is consistent with previous studies showing that 4-HNE-adducts exist in the gastric foveolae of healthy people under physiological condition [58,59]. Production of 4-HNE in other organs merits further investigations but is beyond the scope of this study.

Nonetheless, the strength of this study is the comprehensive analysis of IHC staining for Gsta4, 4-HNE adducts, TNFα, and IL6 by assessing both staining intensity and percentage of positively stained cells. In addition, to the best of our knowledge, this is the first report of analyzing correlations between oxidative markers and inflammatory cytokines in CAC model. Our results show that the expression of oxidative biomarkers is strongly correlated with the expression of inflammatory cytokines in CAC biopsies.

In summary, we show in this study that 4-HNE adducts and Gsta4 are strongly expressed in both epithelial cells and lamina propria in the AOM/DSS-induced CAC model. Gsta4 expression is positively correlated with 4-HNE adducts in the colon biopsies. Furthermore, 4-HNE adducts and Gsta4 are strongly correlated with the expression of inflammatory cytokines TNFα and IL6 in the rectal biopsies. These results demonstrate that oxidative biomarkers Gsta4 and 4-HNE are concurrently upregulated in CAC, supporting the tumor-promoting roles of 4-HNE and Gsta4. Therefore, 4-HNE adducts and Gsta4 expression may have the potential to serve as histopathological biomarkers for CAC and help developing novel preventive strategies for CAC.

## 4 Materials and methods

### 4.1 AOM/DSS-induced CAC model

Animal study was approved by the Institutional Animal Care and Use Committee of Nantong University (Approval number: S20200903-402). Murine CAC model was established using AOM in combination with DSS according to previously reported protocols [32]. Briefly, eight-week-old specific pathogen-free male C57BL/6J mice were randomly divided into two groups, 7 mice per group per time point. All mice were fed with normal diet. For AOM/DSS treatment group, mice were intraperitoneally administered with 10 mg/kg body weight of AOM (Sigma-Aldrich, Shanghai, China) in sterile isotonic saline at day 0, seven days prior to starting DSS treatment. Each DSS treatment cycle was comprised of 7 days of 2.5% DSS (MP Biomedicals, Irvine, CA, USA) in drinking water followed by 14 days of standard drinking water. For the control group, AOM was substituted with saline and standard drinking water was given for all mice throughout the experiment. Mouse health condition, body weight, and hematochezia were monitored twice a week. Following each cycle of DSS treatment, mice were euthanized by cervical dislocation under anaesthetization by isoflurane inhalation and necropsied. Blood samples were collected by cardiac puncture at necropsy and serum isolated for enzyme-linked immunosorbent assay (ELISA). Colon biopsies were fixed with 10% neutral buffered formalin overnight and stored in 70% ethanol for histopathology and immunohistochemistry and remaining tissues were stored at −80 °C for RNA extraction.

### 4.2 Histopathological evaluation

Tissue biopsies were processed and embedded in paraffin using standard protocols. Cross sections were stained with hematoxylin and eosin (H & E) for pathological evaluation. Because AOM/DSS-induced murine CAC exhibits extensive pathological changes from focal inflammation to carcinomas in situ, occasionally, submucosal tumor infiltration depending on the mouse strain, dose of AOM/DSS, and duration of treatment [60,61], pathological changes was evaluated and scored based on the number of inflammatory cells, decrease in goblet cells, reactive atypia/dysplasia, and submucosal tumor infiltration (0, negative; 1, weak; 2, moderate; and 3, strong). These criteria represent major pathological changes for AOM/DSS-induced CAC. Of these, goblet cells are source of mucin that forms intestinal barrier [62], therefore, decrease in the number of goblet cells results in impaired mucus barrier leading to inflammation. While atypia represents benign cellular abnormalities, dysplasia describes neoplastic events [63]. Finally, submucosal tumor infiltration is often relevant to lymphatic metastasis [61,64].

### 4.3 ELISA

Serum Gsta4, 4-HNE, TNFα, and IL6 were quantified using commercially available ELISA kits including Glutathione *S*-Transferase Alpha 4 ELISA kit (USCNK, Wuhan, China), mouse 4-HNE ELISA kit (FineTest, Wuhan, China), mouse TNFα uncoated ELISA kit (ThermoFisher Scientific, Shanghai, China), and mouse IL6 uncoated ELISA kit (ThermoFisher Scientific, Shanghai, China) according to the manufacture's instruction. Mouse serum was diluted with PBS (1:4 for TNFα and IL6 assay; 1:1 for Gsta4 assay; and 1:5 for 4-HNE assay) and 100 μl/well was used for ELISA. Standard curves were created using serially diluted standards included in the assay kits. Absorbance was measured at 450 nm on the Varioskan LUX microplate reader (ThermoFisher Scientific, Waltham, MA, USA) and concentrations were calculated using standard curves.

### 4.4 Real-time quantitative reverse transcriptase PCR (qRT-PCR)

RNA of mouse colons was isolated using the FastPure cell/Tissue Total RNA Isolation Kit V2 (Vazyme, Nanjing, China) and reverse-transcribed into complementary DNA (cDNA) using HiScript III RT SuperMix (Vazyme) according to the manufacturer's instructions. Primers and PCR product for qRT-PCR are listed in Supplementary Table S1. qRT-PCR was carried out using AceQ qPCR SYBR Green Master Mix (Vazyme) by preheating at 95 °C for 5 min followed by 40 cycles at 95 °C for 10s and 60 °C for 30s on the LightCycler 96 Real-Time System (Roche, City, USA).

### 4.5 Immunohistochemistry

Immunohistochemical (IHC) staining for Gsta4, TNFα, IL6, and 4-HNE adducts in the colon biopsies was performed as previously described with minor modification [27]. In brief, tissue sections were deparaffinized and rehydrated. Epitope retrieval for Gsta4, IL6, and 4-HNE adducts was conducted by incubating tissue sections in 100 mM glycine buffer (pH 9.0) at > 97 °C for 10 min. Epitope retrieval for TNFα was carried out by incubating tissue sections in EDTA (pH 9.0) at >97 °C for 10 min. Endogenous peroxidase activity was blocked by sequentially incubating tissue sections with 3% hydrogen peroxide in methanol and 3% hydrogen peroxide in distilled water for 20 min each. Tissue sections were then treated with Pepsin Antigen Retrieval Kit (Sangon Biotech, Shanghai, China) at 37 °C for 30 min. After washing with tris-buffered saline containing 0.1% tween-20 (TBS-T, pH 8.4) for 3 times, tissue sections were blocked with 5% normal goat serum in TBS (pH 8.4) for 1 h and incubated with primary antibody at room temperature overnight. The primary antibodies include rabbit anti-GSTA4 polyclonal antibody (Abnova, CA, USA; 1:50), rabbit anti-TNFα polyclonal antibody (Sangon Biotech; 1:100), rabbit anti-IL6 polyclonal antibody (Affinity, Changzhou, China; 1:2000), and rabbit anti-4-HNE antiserum (Alpha Diagnostic International, Texas, USA; 1:1000). Goat anti-rabbit IgG (H + L)-horseradish peroxidase conjugate (Beyotime, Shanghai, China; 1:100) was used as secondary antibody. Chromogenic color development was performed using DAB Immunohistochemistry Color Development Kit (Sangon Biotech) and nuclei counterstained by hematoxylin (Sangon Biotech) followed by color differentiation using an acid alcohol slow differentiation solution (Beyotime). Immunohistochemical staining for Gsta4, 4-HNE adducts, TNFα, and IL6 was scored using a combination of staining intensity (Scored 0, 1, 2, and 3 for negative, weak, moderate, and strong staining, respectively) and overall number of positive cells per × 10 field (0, none; 1,<10%; 2, 10–50%; and 3, >50% positive cells) as previously described [26].

### 4.6 Statistical analysis

Data are expressed as mean ± standard error of the mean (SEM). All statistical analyses were performed using GraphPad Prism 8.0 (GraphPad Software, California, USA). Unpaired *t*-tests were used for comparisons between experimental and control groups. Correlation analysis was performed using the Pearson correlation coefficient. *P*-value <0.05 was considered statistically significant.

## Ethics statement

Animal study was approved by the Institutional Animal Care and Use Committee of Nantong University (Approval number: S20200903-402).

## Author contribution statement

Chunhua Ma: Conceived and designed the experiments; Performed the experiments; Analyzed and interpreted the data; Wrote the paper.

Zhanhu Zhang: Conceived and designed the experiments; Performed the experiments; Analyzed and interpreted the data.

Tianqi Li; Guoxiang Zhu; Jinyun Zhai: Performed the experiments.

Yumei Tao: Analyzed and interpreted the data.

Lili Xu; Yuanyuan Ju; Xu Huang: Performed the experiments; Contributed reagents, materials, analysis tools or data.

Xingmin Wang: Conceived and designed the experiments; Supervised the research; Analyzed and interpreted the data; Wrote the paper.

## Data availability statement

Data included in article/supplementary material/referenced in article.

## Declaration of competing interest

The authors declare that they have no known competing financial interests or personal relationships that could have appeared to influence the work reported in this paper.
