# Supplementary material for: Colonic expression of glutathione S-transferase alpha 4 and 4-hydroxynonenal adducts is correlated with the pathology of murine colitis-associated cancer
Source: Heliyon. 2023 Sep 4;9(9):e19815. doi: 10.1016/j.heliyon.2023.e19815 (PMC10559223; doi:10.1016/j.heliyon.2023.e19815)
Supplement: Multimedia component 1 [file mmc1.docx]

**Supplementary Figure Legends**

**Supplementary Figure S1.** **AOM rarely induces lesions in liver**. (A) H & E staining for liver biopsy from untreated mouse. (B) H & E staining shows rare hepatocyte necrosis in liver biopsy from AOM-treated mouse 1 week after AOM injection (Blue arrow). Scale bar: 100µm.

**Supplementary Figure S2. AOM/DSS treatment does not cause injury to organs outside of intestines.** (A and B) Representative H & E staining for liver biopsies from mice treated with sham or AOM and 3 cycles of DSS. (C and D) Representative H & E staining for pancreatic biopsies from mouse treated with sham or AOM and 3 cycles of DSS. (E and F) Representative H & E staining for stomach biopsies from mouse treated with sham or AOM and 3 cycles of DSS. Scale bar: 100µm.

**Supplementary Figure S3. Serum Gsta4 and 4-HNE are less associated with the pathology of AOM/DSS-induced CAC.** (A and B) ELISA shows transiently increased serum Gsta4 (A) and TNFα (B) after one cycle treatment with DSS. No increased serum Gsta4 and TNFα are noted after the 2^nd^ and 3^rd^ cycles of DSS treatment. (C) Serum Gsta4 is correlated with TNFα following one cycle treatment with DSS. (D and E) No remarkable change is noted for serum IL6 and 4-HNE throughout the experiment. At least 4 mice per group were used for ELISA experiments based on the availability of serum. ***P* < 0.01 compared to control; ns, not significant.

**Supplementary Figure S4. Correlation analyses for 4-HNE adducts, Gsta4, TNFα, and IL6 in the colon biopsies.** Significant correlations exist between the IHC scores of Gsta4 *vs* 4-HNE adducts (A), Gsta4 *vs* IL6 (B), and 4-HNE adducts *vs* IL6 (C) in the colon biopsies. In contrast, no significant correlations are seen for IHC scores of TNFα *vs* Gsta4 (D) and TNFα *vs* 4-HNE adducts (E) in the colon biopsies.

**Supplementary Figure S5. AOM/DSS treatment does not generate 4-HNE adducts in liver, pancreas, and stomach.** (A and B) IHC staining for 4-HNE adducts in liver biopsies from mouse treated with sham or AOM and 3 cycles of DSS. (C and D) IHC staining for 4-HNE adducts in pancreatic biopsies from mouse treated with sham or AOM and 3 cycles of DSS. (E and F) IHC shows partially positive staining for 4-HNE adducts in the gastric foveolae for both control and AOM/DSS-treated mice. Scale bar: 200µm.
